# Supplementary material for: Modeling glioblastoma heterogeneity as a dynamic network of cell states
Source: Mol Syst Biol. 2021 Sep 16;17(9):e10105. doi: 10.15252/msb.202010105 (PMC8444284; doi:10.15252/msb.202010105)
Supplement: Supplementary file 6 — Source Data for Figure 5 [file MSB-17-e10105-s004.zip › Figure5A_sourcedata/GSEA_3071/hallmarks_stateV.GseaPreranked.1623742546329/gsea_report_for_na_pos_1623742546329.html]

Report for na\_pos 1623742546329 [GSEA]

| GS  follow link to MSigDB | GS DETAILS | SIZE | ES | NES | NOM p-val | FDR q-val | FWER p-val | RANK AT MAX | LEADING EDGE || 1 | HALLMARK\_E2F\_TARGETS | Details ... | 163 | 0.66 | 3.95 | 0.000 | 0.000 | 0.000 | 1179 | tags=74%, list=22%, signal=92% |
| 2 | HALLMARK\_G2M\_CHECKPOINT | Details ... | 140 | 0.63 | 3.66 | 0.000 | 0.000 | 0.000 | 968 | tags=65%, list=18%, signal=77% |
| 3 | HALLMARK\_SPERMATOGENESIS | Details ... | 39 | 0.54 | 2.36 | 0.000 | 0.000 | 0.000 | 1026 | tags=54%, list=19%, signal=66% |
| 4 | HALLMARK\_MITOTIC\_SPINDLE | Details ... | 115 | 0.36 | 2.05 | 0.000 | 0.001 | 0.008 | 956 | tags=38%, list=18%, signal=46% |
| 5 | HALLMARK\_DNA\_REPAIR | Details ... | 66 | 0.40 | 2.01 | 0.000 | 0.001 | 0.010 | 1246 | tags=44%, list=24%, signal=57% |
| 6 | HALLMARK\_KRAS\_SIGNALING\_DN | Details ... | 32 | 0.36 | 1.49 | 0.054 | 0.062 | 0.690 | 1547 | tags=53%, list=29%, signal=75% |
| 7 | HALLMARK\_UV\_RESPONSE\_UP | Details ... | 64 | 0.28 | 1.41 | 0.068 | 0.089 | 0.855 | 1128 | tags=34%, list=21%, signal=43% |
| 8 | HALLMARK\_MYC\_TARGETS\_V1 | Details ... | 109 | 0.25 | 1.39 | 0.057 | 0.086 | 0.882 | 435 | tags=16%, list=8%, signal=17% |
| 9 | HALLMARK\_APICAL\_SURFACE | Details ... | 18 | 0.27 | 0.91 | 0.557 | 0.604 | 1.000 | 570 | tags=22%, list=11%, signal=25% |
Table: Gene sets enriched in phenotype **na **[plain text format]****
